# Clinical outcomes of patients with residual medial osteophytes following mobile bearing unicompartmental knee arthroplasty

**DOI:** 10.1371/journal.pone.0205469

**Published:** 2018-10-11

**Authors:** Boonchana Pongcharoen, Nuttawut Chanalithichai

**Affiliations:** Department of Orthopaedic Surgery, Thammasat University, Pathumthani, Thailand; Consorci Parc de Salut MAR de Barcelona, SPAIN

## Abstract

**Introduction:**

The surgical technique used in unicompartmental knee arthroplasty (UKA) is crucial for achieving good short and long term clinical outcomes. The medial mobile bearing UKA has shown excellent clinical outcomes and survivorship. But release of the medial collateral ligament during entering joint is cause of mobile bearing dislocation in short term outcomes and lateral compartment osteoarthritis may occur in the mid to long term outcomes. Removing all osteophytes at the time of UKA is sometime impossible due to their large size and extend to the inferior part of medial tibial plateau and removing them completely my result in release of the MCL. But no data exist on clinical outcomes in such patients.

**Methods:**

We conducted a prospective study from 2010 to 2015 of patients undergoing mobile bearing UKA and classified them in to two groups: those with (Gp1) and without (Gp2) residual osteophytes. Osteophyte size was measured using Hernborg’s technique. The primary outcomes were pain score, functional score, and knee scores and the presence of reported medial knee pain.

**Results:**

176 patients who underwent 199 mobile bearing UKAs were recruited: Gp1 = 42 patients (46 knees) and Gp2 = 134 patients (153 knees). Residual osteophyte sizes ranged from 2.13–9.42 mm (mean 4.12). The mean Gp1 Gp2 pain score (49.04, 48.92, p = 0.84), functional score (83.75, 84.04, p = 0.83) and knee score (89.86, 98.7, p = 0.0.78) scores were almost identical and no one complained of medial joint pain. Followed up ranged from 2 − 7 years (mean 4.23). No patients were lost to follow up.

**Conclusion:**

The patients with residual osteophytes of length less than 9 mm had good and similar clinical outcomes as patients without residual osteophytes following mobile bearing UKA.

**Level of evidence:**

Level II-2, evidence obtained from well-designed cohort studies or case-control studies, preferably from more than one center or research group.

## Introduction

Surgical technique is important for outcomes in unicompartmental knee arthroplasty (UKA). The medial mobile bearing unicompartmental knee arthroplasty (UKA) has shown excellent clinical outcomes [1–9]. During exposing into the joint, releasing the medial collateral ligament (MCL) is strictly prohibited to prevent overcorrection and resulting mobile bearing dislocation [7–9]. Medial osteophytes are usually small and are easily removed with excision of the adjoining tibial plateau. However, some patients have large osteophytes that extend to the inferior part of medial tibial plateau [10,11] and removing them completely may result in release of the MCL. For this reason, some orthopaedists remove as much of the osteophyte as possible, leaving parts on the medial site of tibia. However, residual osteophytes may be cause of pain and make it difficult for the surgeon to determine the size of the tibial component, alignment of the prosthesis and tension of the MCL. To the best of our best knowledge, no study has determined the clinical outcomes in this group of patients. The purpose of our study reported herein was to determine the clinical outcomes of UKA as a function of presence or absence of residual medial osteophytes.

## Patients and methods

A prospective cohort study was conducted from August 2010 to December 2015 at Thammasat University hospital, Pathumthani, Thailand. The study was approved by the Human Research Ethics Committee of the Faculty of Medicine, Thammasat University (Reg. no: MTU- EC-OT-1-053/61). The inclusion criteria were patients with medial osteoarthritis (OA) of the knee with an Alhback score of 2, 3 and 4 [12], who were older than 40 years of age, with a range of movement (ROM) > 90°, a varus deformity < 25°, and flexion contracture < 20° and underwent a medial mobile bearing UKA (Oxford UKA; Zimmer Biomet, Inc, Warsaw, IN, USA), performed by a single surgeon (BP). All participants signed the informed consent document after giving verbal explanation the study protocol. The exclusion criteria were patients with a diagnosis of spontaneous osteonecrosis of the knee (SPONK), intraoperative anterior cruciate ligament (ACL) insufficiency, inflammatory joint disease, gout, post-traumatic arthritis, and primary PF arthritis.

### Outcome measures

The baseline patient characteristics included age, sex, site, The Knee Society Score (KSS) (knee score, pain score, and functional score) [13], body mass index (BMI), degree of varus deformity, flexion contracture, genu recurvatum, and range of motion were recorded (Table 1).

**Table 1:** Baseline characteristics.

Variable	Group I N = 46 knees	Group II N = 153 knees	P value
Age (year)	64.67±6.93(50–80)	64.39±6.96(50–88)	0.82
Sex (male/female)	5/35	21/115	0.55
Site (right/left)	21/25	68/85	0.24
Pain score (points)	12.31 ± 7.1 (0–20)	12.16 ± 5.1 (0–20)	0.34
Functional score (points)	44.00 ± 6.1 (30–50)	47.22 ± 6.5 (35–65)	0.27
Knee score (points)	34.64±3.40(27–40)	34.68±2.30(27–40)	0.93
Range of motion (°)	118.98±8.39(90–125)	120.06±8.97(90–130)	0.47
Varus deformity (°)	4.71±2.79(0–12)	4.57±3.43(0–15)	0.80
Flexion contracture (°)	5.57±5.35(5–15)	5.15±4.62(5–20)	0.71
Genu recurvatum (°)	0.43±1.69(5–10)	1.12±2.74(5–15)	0.10
BMI (kg/m^2^)	27.03±4.21(20.81–41.62)	26.98±4.45(20–42.22)	0.94

BMI, body mass index

**Fig 1:**
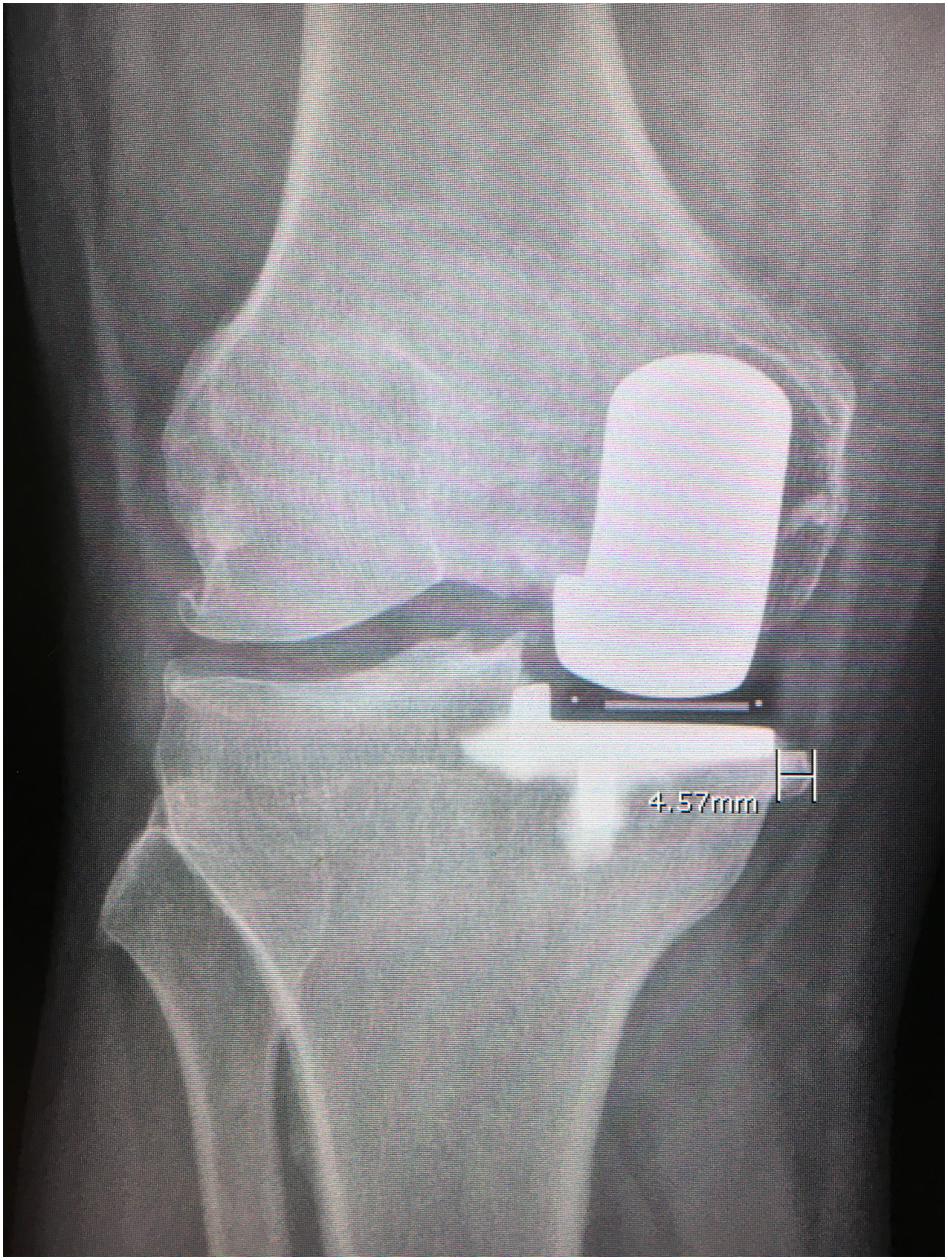
The size of residual osteophyte was measured from the medial cortex of the tibial plateau to the outer margin of osteophyte.

At each follow-up, the patients were also obtained AP standing, lateral standing, skyline view, and long-leg radiographs and recorded the component alignment, and tibiofemoral angle. The Knee Society Score (KSS) (knee score, pain score, and functional score) and the incidence of medial knee pain were also assessed in a blinded fashion by a research assistant at each visit. The lengths of preoperative and residual medial osteophytes were measured using Hernborg’s technique (Fig 1) [11]. The postoperative range of motion was recorded with long arm goniometer. Complications such as infection, component loosening, fractures, and bearing dislocations were recorded.

### Sample size and statistical analysis

The sample-size was calculated based on postoperative KSS, is need to detect a clinical relevant difference of 6 points using standard deviation of 3.0 [14]. Statistics have shown that 40 knees in each group, would have 80% power at the significant 5%.

We determined differences in the age, knee score, pain score, functional score, BMI, ROM, tibiofemoral angle, flexion contractures, genu recurvatum, and VAS for postoperative medial knee pain using the Student’s t test. We determined differences in gender, ratio of sex, ratio of operative site, and incidence of postoperative medial knee pain using Chi squared. All analyses were two sided and a p value of ≤ 0.05 denoted statistical significance.

### Surgical technique

**Fig 2:**
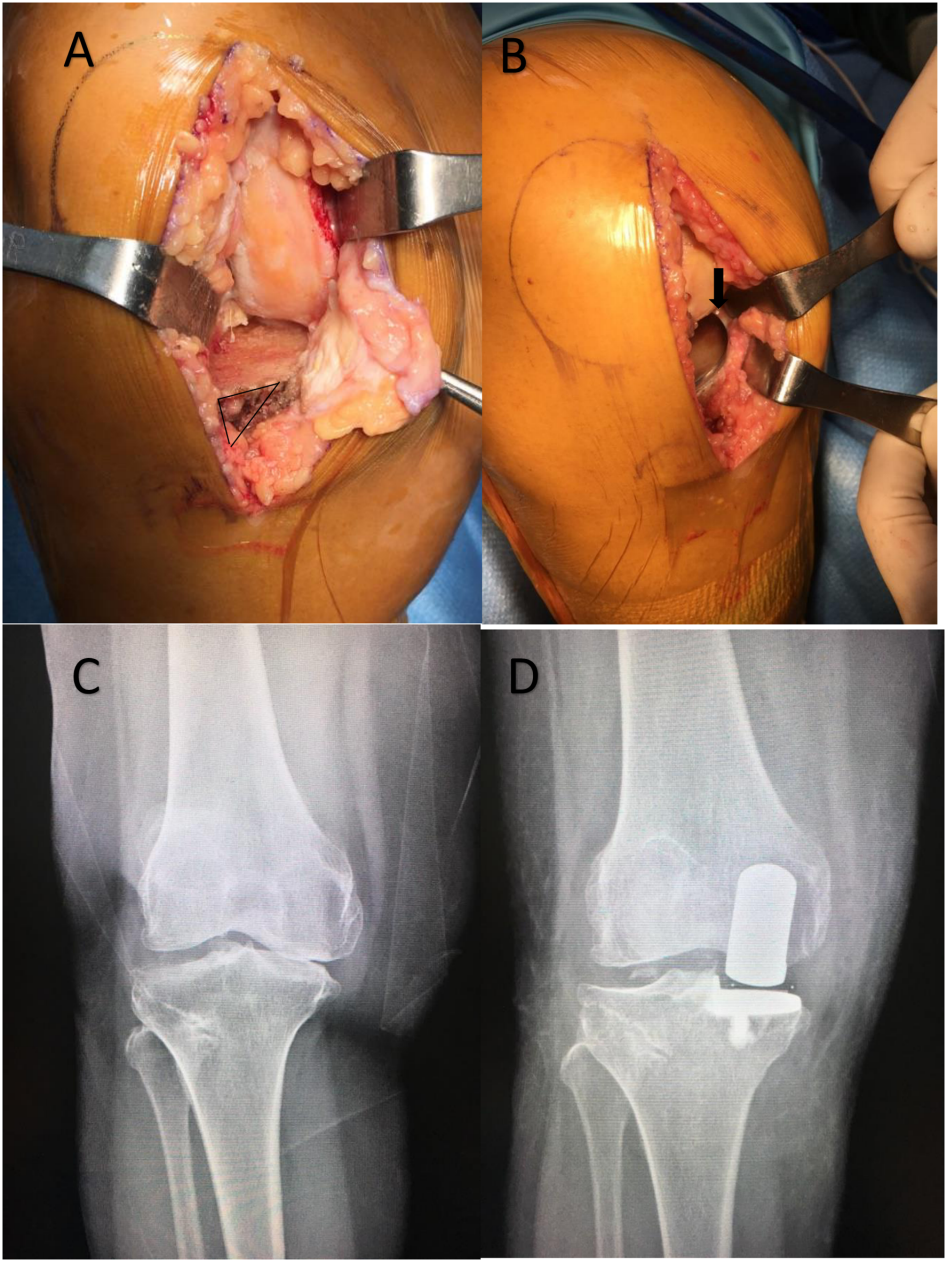
Only the anterior capsule was released when entering the knee joint (triangle shape). Removal of as much of the anteromedial osteophyte was done but always keeping above the insertion of the medial collateral ligament MCL and capsule (A). The anteromedial cortex was identified and used to position of the tibial component to prevent implant overhang. The posteromedial osteophyte (arrow) was difficult to remove without releasing MCL (B). The preoperative radiographic showing the large medial osteophyte (C). The postoperative radiograph (D) showing the residual osteophyte.

The patients were anesthetized via a spinal block with morphine 0.1 mg to 0.2 mg. A thigh tourniquet was inflated to 300 mmHg in all cases prior to the skin incision. All patients received 1 gm cefazolin intravenously before skin incision. An anteromedial skin incision was performed from the upper pole of the patella to the medial aspect of the tibial tubercle. A mini-midvastus approach was applied in all cases. Only the anterior capsule of the proximal tibia was released approximately 1 cm below the joint line, followed by removal of the anterior osteophyte of the proximal tibia (Fig 2A). The patella was slightly subluxated laterally but was not everted and the femoral osteophyte was removed during this step. Minimally invasive instrumentation was used in all cases. The shaft of the tibial saw guide was parallel to the long axis of the tibia to create 7° of tibial slope. The depth of the tibial bone cut was 2 mm below the deepest part of the medial tibial plateau and perpendicular to the mechanical axis. The piece of proximal tibia was used for sizing tibial component. The posterior condyle of the femur was then cut using intramedullary (IM) femoral guided instrumentation that connected the femoral drill guide with the intramedullary (IM) link. The flexion gap was set at 100° of flexion and the extension gap was set at 20° of flexion. The distal condyle of the femur was cut by the milling technique for creating an equal flexion-extension gap. Osteophytes on the anteromedial aspect of tibia were reassessed and removed as much as possible above insertion of capsule and MCL with a bone rongeur and bone osteotome (Fig 2B). The anteromedial part of tibia component was seated on anteromedial aspect of tibia for prevention tibial component overhang (Fig 2B). However, large posteromedial osteophytes extending to the inferior part of proximal tibia (Fig 2B) that could not be excised completely were partially excised (Fig 2D). All components of the implant were assessed before it was fixed. All operations used the same instrumentation. 30 mL of bupivacaine were injected prior to closing the incision. One intraarticular drain (10-gauge) was inserted before closing. The operation time and the presence of intra operative fractures were recorded.

### Postoperative protocol

Patients were taught to perform quadriceps exercises, approximately 20 repetitions, three times daily. They were also instructed to begin ankle pump exercises as soon as possible to reduce the risks of developing a deep vein thrombosis (DVT) and / or pulmonary embolism (PE), and to improve patellar tracking. Patients began ambulation with partial weight bearing and active assisted ROM knee exercises on post-operative Day 1. The patients were discharged from the hospital if the surgical wound was clean and they did not need intravenous pain medication. The patients were followed up at 2 weeks, 6 weeks, 3 months, 6 months, 1 year, and then annually.

## Results

A total of 178 patients (201 knees) were enrolled in this study. Two patients did not complete the study due to a medial tibial plateau fracture at 3 months postoperatively and mobile bearing dislocation at 3 years postoperatively and were excluded from the analysis. The mean follow up was 50.76 months (range 48–90 months) and no patients lose to follow up.

Of the 176 patients (199 knees) included in the final analysis, 42 patients (46 knees) had residual osteophytes, group 1 (Gp1) and 134 patients (153 knees) did not have residual medial osteophytes, Gp2. The preoperative baseline characteristics, including demographic data, KSS, deformity, and ROM, were not significantly different between the two groups (Table 1). Postoperatively, no patients reported post-operative medial knee pain (Table 2). The pain score, knee score, and functional score were not different between two groups (Table 2) and the ROM, knee alignment, prosthesis alignment, and operation time also were not significant different between the two groups (Table 3). The mean length of preoperative medial osteophyte of Gp2 was 1.97±0.95 (range 0.56–4.28) mm. The mean length of preoperative medial osteophyte and retained medial osteophytes of Gp1 were 5.58±2.04 (range 2.01–10.20) mm and 4.12 ±1.67 (range 2.13–9.42) mm., respectively (p<0.001).

**Table 2:** Pain score, functional score, knee score and medial knee pain.

Variable	Group I N = 46 knees	Group II N = 153 knees	P value
Pain score (points)	49.04 ± 2.83 (40–50)	48.92 ± 2.57 (40–50)	0.84
Functional score (points)	83.75 ± 5.74 (80–100)	84.04 ± 6.33 (65–90)	0.83
Knee score (points)	98.86±3.64(81–100)	98.71±3.08(81–100)	0.78
Incidence of postoperative medial knee pain (%)	0	0	NS
VAS for postoperative medial knee (point)	0	0	NS

VAS, visual analog scale

**Table 3:** Secondary outcomes.

Variable	Group I N = 46 knees	Group II N = 153 knees	P value
Range of motion (°)	127.50±5.23 (90–135)	128.45±6.71(90–135)	0.39
Femoral component alignment (°)	Valgus 5.73±2.11(2–8)	Valgus 5.77±1.61(2–10)	0.89
Tibial component alignment (°)	Varus 0.52±1.17 (valgus 2-varus 3)	Varus 0.85±1.02(valgus 1-varus 3)	0.10
Knee alignment (°)	Valgus 4.79±1.85 (varus 2-valgus 7)	Valgus 5.08±1.91(varus 1-valgus 10)	0.38
Operative time (mins)	92.38±10.65 (75–115)	96.06±14.16 (65–120)	0.13

## Discussion

To our knowledge this is the first study to determine the clinical outcome of patients with residual medial osteophytes following medial mobile bearing UKAs. The patients with and without residual medial osteophytes had good clinical outcomes and there were no differences in pain score, functional score, and knee score. Moreover, no patients in either group reported postoperative medial knee pain during follow up.

Patients with medial osteoarthritis (OA) knee usually present with along the medial joint line pain as a result of overloading of subchondral bone, local inflammation and marginal osteophyte formation [15–17]. Why osteophytes cause pain in this area is unclear [18–21]. Osteophyte formation in OA knee is a compensatory mechanism to disease progression and improves stability of knee and increases the area of weight bearing [20,21]. Osteophytes are covered with fibrocartilage and may be seen in knee OA patients who do not report knee pain and so are not thought to cause pain [18,20,21]. Medial knee pain in patients who underwent UKA is caused by overloading on the medial plateau, local inflammation, over hanging of the tibial component, overstretching of the medial collateral ligament from applying too thick of polyethylene, preoperative bone marrow edema, loose bodies, and recurrent hematosis [22–27]. There are no reports cause of pain from MCL stretching due to osteophytes after UKA. However, during the operations, as much of the medial osteophytes were excised without releasing the deep and superficial MCL in this study. The mean size of residual medial osteophytes was just over 4 mm with some osteophytes almost reaching 9 mm. Therefore, most of the anteromedial osteophytes were excised but some deep rooted posteromedial osteophytes were left behind. However, no residual medial osteophyte extended above the tibial component in this study. Five patients in Gp1 had overhanging of the tibial component; the residual osteophytes in these patients measured between 1.38 to 3.13 mm. However, these patients had excellent clinical outcomes and no medial knee pain. LaPrade et al. and Bosania et al. have reported that the proximal insertion of the superficial MCL is a mean distance of 12.2 mm from the joint line [28,29], meaning the insertion point is very close to the joint line in some patients and might be released when entering the joint. Accordingly, we advise against the complete removal of osteophyte because it may result in mobile bearing dislocation and overcorrection.

This study has had some limitations. Firstly, this study was not randomized control trial study; the groups were self-selecting depending on the presence or absence of osteophytes. Despite this, baseline characteristics were similar. Secondly, patients with residual medial osteophytes may have more advanced knee OA and poorer clinical outcomes. However, clinical outcomes were not significantly different between the two groups. Thirdly, the residual medial osteophyte may be the cause of too much under correction which in turn would affect knee alignment. However, mean knee alignments were similar between the two groups (valgus 4.8° Gp1, valgus 5.1° Gp2).

In conclusion, clinical outcomes were good in patients undergoing medial mobile UKA with residual medial osteophytes < 9 mm and were similar to patients without retained medial osteophytes.

## Supporting information

S1 File STROB checklist. (DOC)

S2 File Data. (DOCX)
