# Supplementary material for: Clinical outcomes of patients with residual medial osteophytes following mobile bearing unicompartmental knee arthroplasty
Source: PLoS One. 2018 Oct 11;13(10):e0205469. doi: 10.1371/journal.pone.0205469 (PMC6181378; doi:10.1371/journal.pone.0205469)
Supplement: S2 File — (DOCX) [file pone.0205469.s002.docx]

| patient | group | BMI | Varus deformity | Pre op. ROM | Pain score | Functional score | Pre KSS |
| --- | --- | --- | --- | --- | --- | --- | --- |
| 1 | 1 | 24.44 | 2 | 120 | 10 | 35 | 34 |
| 2 | 1 | 25.71 | 4 | 120 | 10 | 55 | 34 |
| 3 | 1 | 26.66 | 8 | 120 | 20 | 55 | 44 |
| 4 | 1 | 28.88 | 0 | 125 | 10 | 55 | 37 |
| 5 | 1 | 24.44 | 5 | 120 | 10 | 55 | 34 |
| 6 | 1 | 24.44 | 5 | 125 | 10 | 55 | 34 |
| 7 | 1 | 29.38 | 5 | 120 | 10 | 35 | 34 |
| 8 | 1 | 41.62 | 2 | 120 | 10 | 55 | 27 |
| 9 | 1 | 31.11 | 5 | 125 | 20 | 55 | 37 |
| 10 | 1 | 21.4 | 5 | 110 | 10 | 55 | 34 |
| 11 | 1 | 24.44 | 2 | 125 | 20 | 35 | 37 |
| 12 | 1 | 29.13 | 12 | 90 | 20 | 55 | 37 |
| 13 | 1 | 26.44 | 5 | 110 | 10 | 55 | 30 |
| 14 | 1 | 22.89 | 6 | 125 | 10 | 35 | 34 |
| 15 | 1 | 27.05 | 8 | 110 | 10 | 55 | 34 |
| 16 | 1 | 29.38 | 5 | 120 | 10 | 55 | 34 |
| 17 | 1 | 27.34 | 2 | 125 | 20 | 55 | 40 |
| 18 | 1 | 26.44 | 5 | 110 | 10 | 55 | 30 |
| 19 | 1 | 28.88 | 0 | 125 | 20 | 35 | 37 |
| 20 | 1 | 22.95 | 8 | 125 | 10 | 35 | 34 |
| 21 | 1 | 29.38 | 1 | 125 | 10 | 55 | 34 |
| 22 | 1 | 24.44 | 2 | 120 | 10 | 55 | 34 |
| 23 | 1 | 25.71 | 4 | 120 | 10 | 35 | 34 |
| 24 | 1 | 26.66 | 8 | 120 | 20 | 55 | 44 |
| 25 | 1 | 28.88 | 0 | 125 | 20 | 35 | 37 |
| 26 | 1 | 24.44 | 5 | 120 | 10 | 35 | 34 |
| 27 | 1 | 24.44 | 5 | 125 | 10 | 35 | 34 |
| 28 | 1 | 29.38 | 5 | 120 | 10 | 35 | 34 |
| 29 | 1 | 41.62 | 2 | 120 | 10 | 35 | 27 |
| 30 | 1 | 31.11 | 5 | 125 | 20 | 55 | 37 |
| 31 | 1 | 21.4 | 5 | 110 | 10 | 55 | 34 |
| 32 | 1 | 24.44 | 2 | 125 | 10 | 35 | 37 |
| 33 | 1 | 29.13 | 12 | 90 | 20 | 55 | 37 |
| 34 | 1 | 26.44 | 5 | 110 | 20 | 35 | 30 |
| 35 | 1 | 22.89 | 6 | 125 | 10 | 35 | 34 |
| 36 | 1 | 27.05 | 8 | 110 | 10 | 35 | 34 |
| 37 | 1 | 29.38 | 5 | 120 | 10 | 35 | 34 |
| 38 | 1 | 28.88 | 2 | 125 | 10 | 35 | 33 |
| 39 | 1 | 26.44 | 5 | 110 | 10 | 55 | 30 |
| 40 | 1 | 27.54 | 2 | 125 | 10 | 35 | 34 |
| 41 | 1 | 23.52 | 2 | 125 | 10 | 35 | 34 |
| 42 | 1 | 32.87 | 8 | 120 | 20 | 35 | 40 |
| 43 | 1 | 24.44 | 3 | 125 | 10 | 35 | 34 |
| 44 | 1 | 20.81 | 5 | 125 | 10 | 35 | 34 |
| 45 | 1 | 22.22 | 4 | 125 | 10 | 35 | 34 |
| 46 | 1 | 27.05 | 0 | 120 | 10 | 35 | 37 |
| 47 | 2 | 25.77 | 5 | 115 | 20 | 35 | 40 |
| 48 | 2 | 25.71 | 3 | 125 | 20 | 35 | 37 |
| 49 | 2 | 25.95 | 5 | 120 | 20 | 55 | 37 |
| 50 | 2 | 21.4 | 1 | 120 | 20 | 35 | 37 |
| 51 | 2 | 22.95 | 2 | 125 | 10 | 55 | 34 |
| 52 | 2 | 26.44 | 4 | 125 | 10 | 35 | 34 |
| 53 | 2 | 28.88 | 5 | 130 | 20 | 35 | 37 |
| 54 | 2 | 33.49 | 6 | 120 | 10 | 55 | 34 |
| 55 | 2 | 24.44 | 5 | 115 | 20 | 55 | 37 |
| 56 | 2 | 30.91 | 2 | 120 | 10 | 55 | 34 |
| 57 | 2 | 30.91 | 5 | 120 | 10 | 55 | 34 |
| 58 | 2 | 26.54 | 1 | 125 | 20 | 55 | 37 |
| 59 | 2 | 28.88 | 7 | 130 | 10 | 35 | 34 |
| 60 | 2 | 26.66 | 11 | 90 | 10 | 35 | 33 |
| 61 | 2 | 26.44 | 8 | 125 | 10 | 55 | 34 |
| 62 | 2 | 20 | 5 | 130 | 0 | 35 | 27 |
| 63 | 2 | 27.5 | 2 | 125 | 20 | 55 | 37 |
| 64 | 2 | 29.38 | 10 | 125 | 10 | 35 | 30 |
| 65 | 2 | 25.77 | 5 | 125 | 10 | 55 | 34 |
| 66 | 2 | 20.81 | 0 | 130 | 20 | 35 | 37 |
| 67 | 2 | 36.05 | 2 | 125 | 10 | 35 | 37 |
| 68 | 2 | 27.54 | 7 | 100 | 20 | 55 | 37 |
| 69 | 2 | 25.39 | 1 | 125 | 20 | 55 | 37 |
| 70 | 2 | 24.44 | 2 | 125 | 10 | 35 | 37 |
| 71 | 2 | 24.44 | 7 | 125 | 10 | 35 | 34 |
| 72 | 2 | 22.22 | 10 | 125 | 10 | 55 | 34 |
| 73 | 2 | 28.88 | 4 | 125 | 10 | 55 | 34 |
| 74 | 2 | 36.05 | 5 | 125 | 10 | 55 | 37 |
| 75 | 2 | 28.53 | 5 | 120 | 10 | 35 | 34 |
| 76 | 2 | 28.53 | 4 | 120 | 10 | 35 | 34 |
| 77 | 2 | 25.39 | 6 | 120 | 20 | 55 | 37 |
| 78 | 2 | 29.13 | 12 | 90 | 20 | 55 | 37 |
| 79 | 2 | 28.88 | 1 | 130 | 10 | 55 | 37 |
| 80 | 2 | 21.48 | 5 | 120 | 20 | 55 | 37 |
| 81 | 2 | 37.46 | 2 | 115 | 10 | 35 | 34 |
| 82 | 2 | 22.89 | 8 | 125 | 10 | 35 | 34 |
| 83 | 2 | 21.4 | 2 | 110 | 10 | 35 | 37 |
| 84 | 2 | 23.78 | 3 | 120 | 10 | 55 | 34 |
| 85 | 2 | 32.65 | 0 | 115 | 10 | 55 | 34 |
| 86 | 2 | 27.68 | 5 | 125 | 10 | 35 | 34 |
| 87 | 2 | 31.64 | 5 | 120 | 10 | 55 | 34 |
| 88 | 2 | 22.95 | 5 | 125 | 10 | 35 | 34 |
| 89 | 2 | 22.22 | 7 | 125 | 10 | 55 | 34 |
| 90 | 2 | 32.65 | 0 | 115 | 10 | 35 | 34 |
| 91 | 2 | 28.53 | 4 | 120 | 10 | 55 | 34 |
| 92 | 2 | 41.62 | 5 | 120 | 0 | 35 | 27 |
| 93 | 2 | 21.48 | 1 | 120 | 10 | 55 | 34 |
| 94 | 2 | 24.44 | 12 | 125 | 10 | 55 | 34 |
| 95 | 2 | 21.48 | 2 | 125 | 10 | 55 | 34 |
| 96 | 2 | 35.15 | 2 | 110 | 10 | 35 | 34 |
| 97 | 2 | 22.95 | 8 | 125 | 10 | 55 | 34 |
| 98 | 2 | 21.48 | 5 | 120 | 10 | 35 | 34 |
| 99 | 2 | 29.13 | 12 | 90 | 20 | 55 | 37 |
| 100 | 2 | 27.05 | 1 | 125 | 10 | 35 | 34 |
| 101 | 2 | 21.48 | 2 | 125 | 10 | 35 | 34 |
| 102 | 2 | 29.29 | 1 | 125 | 10 | 35 | 33 |
| 103 | 2 | 27.05 | 1 | 125 | 10 | 55 | 34 |
| 104 | 2 | 30.91 | 2 | 125 | 20 | 55 | 40 |
| 105 | 2 | 23.87 | 2 | 125 | 10 | 35 | 34 |
| 106 | 2 | 23.87 | 1 | 125 | 10 | 55 | 34 |
| 107 | 2 | 23.52 | 3 | 130 | 10 | 35 | 34 |
| 108 | 2 | 22.95 | 3 | 125 | 10 | 55 | 34 |
| 109 | 2 | 33.2 | 15 | 125 | 10 | 55 | 34 |
| 110 | 2 | 23.52 | 8 | 125 | 10 | 55 | 34 |
| 111 | 2 | 42.22 | 5 | 90 | 10 | 35 | 34 |
| 112 | 2 | 29.41 | 6 | 125 | 10 | 55 | 34 |
| 113 | 2 | 28.8 | 2 | 100 | 20 | 35 | 37 |
| 114 | 2 | 26.44 | 2 | 115 | 20 | 35 | 37 |
| 115 | 2 | 29.29 | 9 | 115 | 10 | 55 | 33 |
| 116 | 2 | 24.44 | 15 | 110 | 10 | 35 | 30 |
| 117 | 2 | 25.77 | 3 | 125 | 10 | 35 | 34 |
| 118 | 2 | 28.88 | 2 | 125 | 10 | 35 | 33 |
| 119 | 2 | 26.44 | 5 | 110 | 10 | 55 | 30 |
| 120 | 2 | 27.54 | 2 | 125 | 10 | 55 | 34 |
| 121 | 2 | 23.52 | 2 | 125 | 10 | 55 | 34 |
| 122 | 2 | 32.87 | 8 | 120 | 20 | 35 | 40 |
| 123 | 2 | 24.44 | 3 | 125 | 10 | 35 | 34 |
| 124 | 2 | 26.66 | 10 | 115 | 10 | 55 | 33 |
| 125 | 2 | 22.89 | 3 | 120 | 10 | 35 | 34 |
| 126 | 2 | 26.66 | 8 | 120 | 10 | 55 | 33 |
| 127 | 2 | 25.77 | 5 | 120 | 10 | 35 | 33 |
| 128 | 2 | 28.88 | 4 | 120 | 20 | 35 | 36 |
| 129 | 2 | 23.52 | 5 | 125 | 10 | 55 | 34 |
| 130 | 2 | 22.89 | 3 | 125 | 10 | 35 | 34 |
| 131 | 2 | 24.44 | 6 | 125 | 10 | 55 | 33 |
| 132 | 2 | 20.81 | 5 | 125 | 10 | 35 | 34 |
| 133 | 2 | 28.88 | 13 | 120 | 10 | 55 | 34 |
| 134 | 2 | 24.44 | 6 | 125 | 10 | 35 | 34 |
| 135 | 2 | 23.52 | 1 | 120 | 20 | 55 | 40 |
| 136 | 2 | 26.44 | 2 | 120 | 10 | 55 | 34 |
| 137 | 2 | 29.38 | 1 | 125 | 10 | 55 | 34 |
| 138 | 2 | 27.05 | 10 | 90 | 10 | 35 | 34 |
| 139 | 2 | 26.44 | 15 | 110 | 10 | 35 | 33 |
| 140 | 2 | 26.66 | 2 | 125 | 20 | 55 | 40 |
| 141 | 2 | 33.71 | 3 | 120 | 10 | 35 | 30 |
| 142 | 2 | 24.44 | 4 | 110 | 10 | 35 | 34 |
| 143 | 2 | 26.66 | 10 | 115 | 10 | 55 | 33 |
| 144 | 2 | 22.22 | 4 | 125 | 10 | 35 | 34 |
| 145 | 2 | 27.05 | 0 | 120 | 20 | 55 | 37 |
| 146 | 2 | 25.77 | 5 | 115 | 20 | 35 | 40 |
| 147 | 2 | 25.71 | 3 | 125 | 10 | 55 | 37 |
| 148 | 2 | 25.95 | 5 | 120 | 20 | 55 | 37 |
| 149 | 2 | 21.4 | 1 | 120 | 20 | 55 | 37 |
| 150 | 2 | 22.95 | 2 | 125 | 10 | 35 | 34 |
| 151 | 2 | 26.44 | 4 | 125 | 10 | 55 | 34 |
| 152 | 2 | 28.88 | 5 | 130 | 20 | 35 | 37 |
| 153 | 2 | 33.49 | 6 | 120 | 10 | 55 | 34 |
| 154 | 2 | 24.44 | 5 | 115 | 20 | 55 | 37 |
| 155 | 2 | 30.91 | 2 | 120 | 10 | 55 | 34 |
| 156 | 2 | 30.91 | 5 | 120 | 10 | 55 | 34 |
| 157 | 2 | 26.54 | 1 | 125 | 20 | 55 | 37 |
| 158 | 2 | 28.88 | 7 | 130 | 10 | 35 | 34 |
| 159 | 2 | 26.66 | 11 | 90 | 10 | 35 | 33 |
| 160 | 2 | 26.44 | 8 | 125 | 10 | 55 | 34 |
| 161 | 2 | 20 | 5 | 130 | 0 | 55 | 27 |
| 162 | 2 | 27.5 | 2 | 125 | 20 | 55 | 37 |
| 163 | 2 | 29.38 | 10 | 125 | 10 | 55 | 30 |
| 164 | 2 | 25.77 | 5 | 125 | 10 | 35 | 34 |
| 165 | 2 | 20.81 | 0 | 130 | 20 | 55 | 37 |
| 166 | 2 | 36.05 | 2 | 125 | 20 | 55 | 37 |
| 167 | 2 | 27.54 | 7 | 100 | 10 | 35 | 37 |
| 168 | 2 | 25.71 | 1 | 125 | 10 | 55 | 37 |
| 169 | 2 | 24.44 | 2 | 125 | 20 | 55 | 37 |
| 170 | 2 | 24.34 | 7 | 125 | 10 | 55 | 34 |
| 171 | 2 | 22.22 | 10 | 125 | 10 | 35 | 34 |
| 172 | 2 | 28.88 | 4 | 125 | 10 | 55 | 34 |
| 173 | 2 | 36.05 | 5 | 125 | 10 | 55 | 37 |
| 174 | 2 | 28.53 | 5 | 120 | 10 | 35 | 34 |
| 175 | 2 | 28.53 | 4 | 120 | 10 | 35 | 34 |
| 176 | 2 | 25.39 | 6 | 120 | 20 | 55 | 37 |
| 177 | 2 | 29.13 | 12 | 90 | 10 | 55 | 37 |
| 178 | 2 | 28.88 | 1 | 130 | 20 | 55 | 37 |
| 179 | 2 | 21.48 | 5 | 120 | 10 | 55 | 37 |
| 180 | 2 | 37.46 | 2 | 115 | 10 | 35 | 34 |
| 181 | 2 | 22.89 | 8 | 125 | 10 | 55 | 34 |
| 182 | 2 | 21.4 | 2 | 110 | 20 | 55 | 37 |
| 183 | 2 | 23.78 | 3 | 120 | 10 | 55 | 34 |
| 184 | 2 | 32.65 | 0 | 115 | 10 | 55 | 34 |
| 185 | 2 | 27.68 | 5 | 125 | 10 | 55 | 34 |
| 186 | 2 | 31.64 | 5 | 120 | 10 | 55 | 34 |
| 187 | 2 | 22.95 | 5 | 125 | 10 | 55 | 34 |
| 188 | 2 | 22.22 | 7 | 125 | 10 | 55 | 34 |
| 189 | 2 | 32.65 | 0 | 115 | 10 | 55 | 34 |
| 190 | 2 | 28.53 | 4 | 120 | 10 | 55 | 34 |
| 191 | 2 | 41.62 | 5 | 120 | 0 | 55 | 27 |
| 192 | 2 | 21.48 | 1 | 120 | 10 | 35 | 34 |
| 193 | 2 | 24.44 | 12 | 125 | 10 | 35 | 34 |
| 194 | 2 | 21.48 | 2 | 125 | 10 | 55 | 34 |
| 195 | 2 | 35.15 | 2 | 110 | 10 | 55 | 34 |
| 195 | 2 | 22.95 | 8 | 125 | 10 | 35 | 34 |
| 197 | 2 | 21.48 | 5 | 120 | 10 | 55 | 34 |
| 198 | 2 | 29.13 | 12 | 90 | 20 | 35 | 37 |
| 199 | 2 | 27.05 | 1 | 125 | 10 | 55 | 34 |

| patient | Post pain score | Post functional score | Post KSS | Post ROM | Pre op length of osteophyte | Post op length of osteophyte | Operative time | Knee alignment |
| --- | --- | --- | --- | --- | --- | --- | --- | --- |
| 1 | 45 | 90 | 88 | 120 | 2.13 | 2.01 | 85 | 1 |
| 2 | 50 | 90 | 100 | 130 | 3.7 | 3.49 | 85 | 3 |
| 3 | 40 | 90 | 87 | 125 | 5.83 | 8.02 | 100 | 2 |
| 4 | 50 | 90 | 100 | 130 | 3.86 | 5.11 | 90 | 6 |
| 5 | 50 | 80 | 100 | 125 | 5.24 | 5.5 | 115 | 8 |
| 6 | 50 | 80 | 100 | 130 | 3.14 | 5.18 | 95 | 3 |
| 7 | 50 | 80 | 100 | 130 | 4.91 | 6.26 | 75 | 7 |
| 8 | 50 | 90 | 100 | 125 | 2.86 | 4.52 | 105 | 6 |
| 9 | 50 | 90 | 100 | 130 | 3.54 | 3.94 | 75 | 5 |
| 10 | 50 | 80 | 100 | 120 | 2.92 | 3.49 | 75 | 4 |
| 11 | 50 | 90 | 100 | 130 | 2.65 | 3.75 | 100 | 7 |
| 12 | 50 | 90 | 100 | 110 | 3.93 | 4.54 | 95 | 4 |
| 13 | 50 | 90 | 100 | 130 | 2.16 | 3.81 | 100 | 5 |
| 14 | 50 | 90 | 100 | 130 | 2.68 | 3.46 | 90 | 3 |
| 15 | 50 | 90 | 100 | 125 | 3.38 | 5.74 | 75 | 4 |
| 16 | 50 | 90 | 100 | 130 | 4.29 | 4.72 | 100 | 7 |
| 17 | 50 | 90 | 100 | 130 | 3.51 | 4.1 | 90 | 5 |
| 18 | 50 | 90 | 100 | 130 | 2.47 | 3.1 | 90 | 5 |
| 19 | 50 | 90 | 100 | 130 | 2.8 | 5.11 | 90 | 6 |
| 20 | 50 | 80 | 100 | 135 | 2.16 | 3.1 | 90 | 4 |
| 21 | 50 | 90 | 100 | 130 | 2.96 | 3.1 | 100 | 6 |
| 22 | 40 | 65 | 88 | 120 | 3.7 | 7.67 | 85 | 1 |
| 23 | 50 | 90 | 100 | 130 | 4.76 | 6.3 | 85 | 3 |
| 24 | 40 | 80 | 87 | 125 | 3.76 | 5.69 | 85 | 2 |
| 25 | 50 | 90 | 100 | 130 | 2.56 | 3.46 | 90 | 6 |
| 26 | 50 | 90 | 100 | 125 | 4.48 | 7.33 | 115 | 8 |
| 27 | 50 | 90 | 100 | 130 | 8.87 | 10.2 | 95 | 3 |
| 28 | 50 | 90 | 100 | 130 | 4.36 | 9 | 75 | 7 |
| 29 | 50 | 90 | 100 | 125 | 5.22 | 5.54 | 105 | 6 |
| 30 | 50 | 80 | 100 | 130 | 5.48 | 7.5 | 75 | 5 |
| 31 | 50 | 90 | 100 | 120 | 3.34 | 5.48 | 100 | 4 |
| 32 | 50 | 90 | 100 | 130 | 3.98 | 4.87 | 90 | 7 |
| 33 | 50 | 80 | 100 | 110 | 7.37 | 7.9 | 95 | 4 |
| 34 | 50 | 80 | 100 | 130 | 3.29 | 7.05 | 100 | 5 |
| 35 | 50 | 80 | 100 | 130 | 9.42 | 10.1 | 90 | 3 |
| 36 | 50 | 80 | 100 | 125 | 5.05 | 5.31 | 100 | 4 |
| 37 | 50 | 90 | 100 | 130 | 2.32 | 3.34 | 100 | 7 |
| 38 | 50 | 80 | 100 | 130 | 2.78 | 6.63 | 85 | 8 |
| 39 | 50 | 90 | 100 | 130 | 3.38 | 10.19 | 100 | 5 |
| 40 | 50 | 90 | 100 | 135 | 7.02 | 7.5 | 90 | 6 |
| 41 | 50 | 90 | 100 | 130 | 4.36 | 4.44 | 100 | 3 |
| 42 | 50 | 90 | 100 | 130 | 5.22 | 5.54 | 110 | 4 |
| 43 | 50 | 90 | 100 | 130 | 5.48 | 7.5 | 100 | 3 |
| 44 | 50 | 80 | 100 | 130 | 3.34 | 4.87 | 75 | 6 |
| 45 | 50 | 80 | 92 | 120 | 1.75 | 0 | 105 | 2 |
| 46 | 50 | 80 | 100 | 130 | 2.46 | 0 | 90 | 4 |
| 47 | 50 | 80 | 92 | 120 | 1.44 | 0 | 120 | 7 |
| 48 | 50 | 90 | 100 | 135 | 2.12 | 0 | 115 | 6 |
| 49 | 50 | 90 | 100 | 125 | 1.39 | 0 | 90 | 5 |
| 50 | 50 | 90 | 100 | 130 | 1.7 | 0 | 90 | 8 |
| 51 | 50 | 90 | 100 | 135 | 2.68 | 0 | 120 | 6 |
| 52 | 45 | 80 | 93 | 130 | 2.86 | 0 | 100 | 3 |
| 53 | 50 | 90 | 100 | 140 | 1.1 | 0 | 75 | 7 |
| 54 | 50 | 90 | 100 | 130 | 3.21 | 0 | 105 | 6 |
| 55 | 50 | 90 | 100 | 125 | 2.1 | 0 | 120 | 5 |
| 56 | 50 | 90 | 100 | 130 | 3.95 | 0 | 115 | 2 |
| 57 | 50 | 80 | 100 | 130 | 1.95 | 0 | 100 | 2 |
| 58 | 50 | 80 | 100 | 130 | 1.75 | 0 | 120 | 4 |
| 59 | 50 | 90 | 100 | 130 | 4.18 | 0 | 105 | 2 |
| 60 | 45 | 80 | 92 | 115 | 3.39 | 0 | 120 | 4 |
| 61 | 50 | 90 | 100 | 130 | 2.03 | 0 | 90 | 1 |
| 62 | 45 | 80 | 87 | 130 | 1.75 | 0 | 115 | 7 |
| 63 | 50 | 90 | 100 | 135 | 2.16 | 0 | 85 | 4 |
| 64 | 50 | 90 | 100 | 130 | 4.28 | 0 | 90 | 3 |
| 65 | 50 | 90 | 100 | 135 | 3.09 | 0 | 75 | 6 |
| 66 | 50 | 90 | 100 | 145 | 2 | 0 | 90 | 9 |
| 67 | 50 | 80 | 100 | 130 | 1.52 | 0 | 85 | 6 |
| 68 | 50 | 80 | 100 | 125 | 2.4 | 0 | 90 | 9 |
| 69 | 50 | 90 | 100 | 130 | 1.39 | 0 | 90 | 4 |
| 70 | 50 | 80 | 100 | 130 | 2.16 | 0 | 90 | 7 |
| 71 | 50 | 90 | 100 | 130 | 1.75 | 0 | 95 | 4 |
| 72 | 50 | 80 | 100 | 130 | 1.39 | 0 | 110 | 4 |
| 73 | 50 | 90 | 100 | 130 | 2.16 | 0 | 90 | 4 |
| 74 | 50 | 90 | 100 | 130 | 1.75 | 0 | 100 | 6 |
| 75 | 50 | 90 | 100 | 130 | 1.39 | 0 | 75 | 2 |
| 76 | 50 | 90 | 100 | 130 | 2.16 | 0 | 75 | 4 |
| 77 | 50 | 80 | 100 | 130 | 1.75 | 0 | 100 | 2 |
| 78 | 50 | 90 | 100 | 110 | 1.39 | 0 | 95 | 4 |
| 79 | 50 | 80 | 100 | 140 | 2.16 | 0 | 75 | 7 |
| 80 | 50 | 90 | 100 | 125 | 1.75 | 0 | 95 | 6 |
| 81 | 45 | 80 | 92 | 120 | 1.39 | 0 | 115 | 2 |
| 82 | 50 | 90 | 100 | 130 | 0.95 | 0 | 80 | 3 |
| 83 | 45 | 80 | 92 | 115 | 1.05 | 0 | 80 | 7 |
| 84 | 50 | 90 | 100 | 130 | 0.95 | 0 | 115 | 2 |
| 85 | 50 | 80 | 100 | 125 | 2.57 | 0 | 115 | 8 |
| 86 | 50 | 90 | 100 | 135 | 2.11 | 0 | 90 | 2 |
| 87 | 50 | 90 | 100 | 135 | 2.86 | 0 | 90 | 8 |
| 88 | 50 | 90 | 100 | 135 | 2.11 | 0 | 120 | 6 |
| 89 | 50 | 90 | 100 | 130 | 3.21 | 0 | 85 | 4 |
| 90 | 50 | 80 | 100 | 125 | 3.18 | 0 | 80 | 8 |
| 91 | 50 | 90 | 100 | 130 | 1.23 | 0 | 75 | 4 |
| 92 | 50 | 80 | 100 | 125 | 2.14 | 0 | 105 | 6 |
| 93 | 50 | 90 | 100 | 130 | 1.99 | 0 | 90 | 5 |
| 94 | 50 | 80 | 100 | 135 | 3.68 | 0 | 105 | 7 |
| 95 | 50 | 90 | 100 | 135 | 2.52 | 0 | 105 | 7 |
| 96 | 45 | 80 | 92 | 120 | 1.35 | 0 | 120 | 7 |
| 97 | 50 | 90 | 100 | 135 | 3.45 | 0 | 90 | 4 |
| 98 | 45 | 80 | 92 | 110 | 2.79 | 0 | 120 | 6 |
| 99 | 50 | 90 | 100 | 110 | 2.84 | 0 | 95 | 4 |
| 100 | 50 | 80 | 100 | 130 | 3.66 | 0 | 110 | 6 |
| 101 | 50 | 90 | 100 | 135 | 4.25 | 0 | 120 | 7 |
| 102 | 50 | 80 | 100 | 135 | 2.12 | 0 | 90 | 6 |
| 103 | 50 | 90 | 100 | 130 | 2 | 0 | 75 | 5 |
| 104 | 50 | 90 | 100 | 130 | 1.38 | 0 | 80 | 6 |
| 105 | 50 | 90 | 100 | 130 | 2.74 | 0 | 105 | 4 |
| 106 | 50 | 90 | 100 | 130 | 1.31 | 0 | 75 | 6 |
| 107 | 50 | 80 | 100 | 145 | 1.75 | 0 | 105 | 6 |
| 108 | 50 | 90 | 100 | 130 | 2.75 | 0 | 65 | 6 |
| 109 | 50 | 80 | 100 | 130 | 3.86 | 0 | 105 | 4 |
| 110 | 50 | 90 | 100 | 130 | 1.34 | 0 | 105 | 7 |
| 111 | 45 | 80 | 92 | 120 | 0.5 | 0 | 115 | 6 |
| 112 | 50 | 90 | 100 | 130 | 2.68 | 0 | 105 | 6 |
| 113 | 45 | 80 | 92 | 115 | 1.49 | 0 | 90 | 6 |
| 114 | 50 | 90 | 100 | 125 | 0.5 | 0 | 90 | 5 |
| 115 | 50 | 80 | 100 | 125 | 2.79 | 0 | 80 | 6 |
| 116 | 50 | 90 | 100 | 125 | 2.46 | 0 | 80 | 4 |
| 117 | 50 | 80 | 100 | 130 | 1.02 | 0 | 80 | 6 |
| 118 | 50 | 90 | 100 | 130 | 1.97 | 0 | 85 | 8 |
| 119 | 50 | 90 | 100 | 130 | 1.99 | 0 | 90 | 5 |
| 120 | 50 | 90 | 100 | 135 | 1.1 | 0 | 90 | 6 |
| 121 | 50 | 90 | 100 | 130 | 2.72 | 0 | 100 | 3 |
| 122 | 50 | 80 | 100 | 130 | 2.71 | 0 | 100 | 4 |
| 123 | 50 | 90 | 100 | 130 | 0.5 | 0 | 100 | 3 |
| 124 | 50 | 80 | 100 | 125 | 1.56 | 0 | 90 | 2 |
| 125 | 50 | 90 | 100 | 130 | 0.5 | 0 | 80 | 3 |
| 126 | 50 | 80 | 100 | 130 | 1.7 | 0 | 90 | 6 |
| 127 | 50 | 90 | 100 | 130 | 2.16 | 0 | 80 | 6 |
| 128 | 50 | 80 | 100 | 130 | 1.74 | 0 | 110 | 4 |
| 129 | 50 | 90 | 100 | 130 | 0.5 | 0 | 100 | 5 |
| 130 | 50 | 90 | 100 | 130 | 1.9 | 0 | 80 | 5 |
| 131 | 50 | 90 | 100 | 130 | 1.7 | 0 | 90 | 5 |
| 132 | 50 | 90 | 100 | 130 | 0.5 | 0 | 75 | 6 |
| 133 | 50 | 80 | 100 | 120 | 1.74 | 0 | 100 | 5 |
| 134 | 50 | 90 | 100 | 130 | 1.81 | 0 | 90 | 6 |
| 135 | 50 | 80 | 100 | 130 | 2.44 | 0 | 85 | 8 |
| 136 | 50 | 90 | 100 | 130 | 4.1 | 0 | 85 | 7 |
| 137 | 50 | 90 | 100 | 130 | 3.52 | 0 | 80 | 6 |
| 138 | 45 | 80 | 92 | 115 | 3.72 | 0 | 85 | 6 |
| 139 | 45 | 80 | 92 | 115 | 1.8 | 0 | 100 | 6 |
| 140 | 50 | 90 | 100 | 130 | 1.44 | 0 | 90 | 6 |
| 141 | 45 | 80 | 92 | 120 | 0.6 | 0 | 90 | 6 |
| 142 | 45 | 80 | 92 | 120 | 1.82 | 0 | 100 | 6 |
| 143 | 50 | 90 | 100 | 125 | 2.58 | 0 | 90 | 2 |
| 144 | 45 | 80 | 92 | 120 | 4.2 | 0 | 105 | 2 |
| 145 | 50 | 90 | 100 | 130 | 1.44 | 0 | 90 | 4 |
| 146 | 45 | 80 | 92 | 120 | 2.14 | 0 | 120 | 7 |
| 147 | 50 | 80 | 100 | 135 | 1.26 | 0 | 115 | 6 |
| 148 | 50 | 90 | 100 | 125 | 0.75 | 0 | 90 | 5 |
| 149 | 50 | 80 | 100 | 130 | 1.27 | 0 | 90 | 8 |
| 150 | 50 | 90 | 100 | 135 | 1.38 | 0 | 120 | 6 |
| 151 | 45 | 80 | 93 | 130 | 1.39 | 0 | 100 | 3 |
| 152 | 50 | 90 | 100 | 140 | 2.58 | 0 | 75 | 7 |
| 153 | 50 | 80 | 100 | 130 | 0.8 | 0 | 105 | 6 |
| 154 | 50 | 90 | 100 | 125 | 1.76 | 0 | 120 | 5 |
| 155 | 50 | 80 | 100 | 130 | 2.08 | 0 | 115 | 2 |
| 156 | 50 | 90 | 100 | 130 | 2.12 | 0 | 100 | 2 |
| 157 | 50 | 90 | 100 | 130 | 0.5 | 0 | 120 | 4 |
| 158 | 50 | 90 | 100 | 130 | 1.27 | 0 | 105 | 2 |
| 159 | 45 | 80 | 92 | 115 | 1.57 | 0 | 120 | 4 |
| 160 | 50 | 90 | 100 | 130 | 0.5 | 0 | 90 | 1 |
| 161 | 45 | 80 | 87 | 130 | 2.63 | 0 | 115 | 7 |
| 162 | 50 | 90 | 100 | 135 | 1.84 | 0 | 85 | 4 |
| 163 | 50 | 80 | 100 | 130 | 1.18 | 0 | 90 | 3 |
| 164 | 50 | 90 | 100 | 135 | 0.5 | 0 | 75 | 6 |
| 165 | 50 | 80 | 100 | 145 | 1.2 | 0 | 90 | 9 |
| 166 | 50 | 90 | 100 | 130 | 3.42 | 0 | 85 | 6 |
| 167 | 50 | 90 | 100 | 125 | 1.27 | 0 | 90 | 9 |
| 168 | 50 | 90 | 100 | 130 | 1.7 | 0 | 90 | 4 |
| 169 | 50 | 90 | 100 | 130 | 1.34 | 0 | 90 | 7 |
| 170 | 50 | 80 | 100 | 130 | 2.61 | 0 | 95 | 4 |
| 171 | 50 | 90 | 100 | 130 | 3.11 | 0 | 110 | 4 |
| 172 | 50 | 80 | 100 | 130 | 1.05 | 0 | 90 | 4 |
| 173 | 50 | 90 | 100 | 130 | 0.75 | 0 | 100 | 6 |
| 174 | 50 | 90 | 100 | 130 | 1.25 | 0 | 75 | 2 |
| 175 | 50 | 90 | 100 | 130 | 2.16 | 0 | 75 | 4 |
| 176 | 50 | 90 | 100 | 130 | 1.27 | 0 | 100 | 2 |
| 177 | 50 | 80 | 100 | 110 | 3.52 | 0 | 95 | 4 |
| 178 | 50 | 90 | 100 | 140 | 1.41 | 0 | 75 | 7 |
| 179 | 50 | 80 | 100 | 125 | 1.63 | 0 | 95 | 6 |
| 180 | 45 | 80 | 92 | 120 | 3.28 | 0 | 115 | 2 |
| 181 | 50 | 90 | 100 | 130 | 3.75 | 0 | 80 | 3 |
| 182 | 45 | 80 | 92 | 115 | 2.12 | 0 | 80 | 7 |
| 183 | 50 | 90 | 100 | 130 | 2.01 | 0 | 115 | 2 |
| 184 | 50 | 90 | 100 | 125 | 3.14 | 0 | 115 | 8 |
| 185 | 50 | 90 | 100 | 135 | 1.62 | 0 | 90 | 2 |
| 186 | 50 | 90 | 100 | 135 | 0.5 | 0 | 90 | 8 |
| 187 | 50 | 80 | 100 | 135 | 2.12 | 0 | 120 | 6 |
| 188 | 50 | 90 | 100 | 130 | 0.85 | 0 | 85 | 4 |
| 189 | 50 | 80 | 100 | 125 | 0.46 | 0 | 80 | 8 |
| 190 | 50 | 90 | 100 | 130 | 0.91 | 0 | 75 | 4 |
| 191 | 50 | 90 | 100 | 125 | 1.31 | 0 | 105 | 6 |
| 192 | 50 | 90 | 100 | 130 | 0.73 | 0 | 90 | 5 |
| 193 | 50 | 90 | 100 | 135 | 3.83 | 0 | 105 | 7 |
| 194 | 50 | 90 | 100 | 135 | 3.21 | 0 | 105 | 7 |
| 195 | 45 | 80 | 92 | 120 | 1.7 | 0 | 120 | 7 |
| 195 | 50 | 90 | 100 | 135 | 0.94 | 0 | 90 | 4 |
| 197 | 45 | 80 | 92 | 110 | 0.54 | 0 | 120 | 6 |
| 198 | 50 | 90 | 100 | 110 | 1.6 | 0 | 95 | 4 |
| 199 | 50 | 90 | 100 | 130 | 2.12 | 0 | 110 | 6 |

| patient | Femoral component alignment | Tibial component alignment |  |  |  |  |  |  |  |
| --- | --- | --- | --- | --- | --- | --- | --- | --- | --- |
| 1 | 10 | 3 |  |  |  |  |  |  |  |
| 2 | 7 | 0 |  |  |  |  |  |  |  |
| 3 | 4 | 1 |  |  |  |  |  |  |  |
| 4 | 5 | 0 |  |  |  |  |  |  |  |
| 5 | 6 | -1 |  |  |  |  |  |  |  |
| 6 | 7 | -2 |  |  |  |  |  |  |  |
| 7 | 2 | 1 |  |  |  |  |  |  |  |
| 8 | 9 | 0 |  |  |  |  |  |  |  |
| 9 | 4 | -1 |  |  |  |  |  |  |  |
| 10 | 2 | 0 |  |  |  |  |  |  |  |
| 11 | 6 | 2 |  |  |  |  |  |  |  |
| 12 | 8 | 2 |  |  |  |  |  |  |  |
| 13 | 5 | 0 |  |  |  |  |  |  |  |
| 14 | 8 | 0 |  |  |  |  |  |  |  |
| 15 | 6 | 0 |  |  |  |  |  |  |  |
| 16 | 2 | 1 |  |  |  |  |  |  |  |
| 17 | 6 | 0 |  |  |  |  |  |  |  |
| 18 | 5 | 0 |  |  |  |  |  |  |  |
| 19 | 5 | 0 |  |  |  |  |  |  |  |
| 20 | 6 | 2 |  |  |  |  |  |  |  |
| 21 | 7 | 1 |  |  |  |  |  |  |  |
| 22 | 10 | 3 |  |  |  |  |  |  |  |
| 23 | 7 | 0 |  |  |  |  |  |  |  |
| 24 | 4 | 1 |  |  |  |  |  |  |  |
| 25 | 5 | 0 |  |  |  |  |  |  |  |
| 26 | 6 | -1 |  |  |  |  |  |  |  |
| 27 | 7 | -2 |  |  |  |  |  |  |  |
| 28 | 2 | 1 |  |  |  |  |  |  |  |
| 29 | 9 | 0 |  |  |  |  |  |  |  |
| 30 | 4 | -1 |  |  |  |  |  |  |  |
| 31 | 2 | 0 |  |  |  |  |  |  |  |
| 32 | 6 | 2 |  |  |  |  |  |  |  |
| 33 | 8 | 2 |  |  |  |  |  |  |  |
| 34 | 5 | 0 |  |  |  |  |  |  |  |
| 35 | 8 | 0 |  |  |  |  |  |  |  |
| 36 | 6 | 0 |  |  |  |  |  |  |  |
| 37 | 2 | 1 |  |  |  |  |  |  |  |
| 38 | 7 | 2 |  |  |  |  |  |  |  |
| 39 | 5 | 0 |  |  |  |  |  |  |  |
| 40 | 7 | 0 |  |  |  |  |  |  |  |
| 41 | 6 | 1 |  |  |  |  |  |  |  |
| 42 | 5 | 1 |  |  |  |  |  |  |  |
| 43 | 6 | 2 |  |  |  |  |  |  |  |
| 44 | 5 | 2 |  |  |  |  |  |  |  |
| 45 | 10 | 3 |  |  |  |  |  |  |  |
| 46 | 6 | 1 |  |  |  |  |  |  |  |
| 47 | 4 | 2 |  |  |  |  |  |  |  |
| 48 | 8 | -1 |  |  |  |  |  |  |  |
| 49 | 6 | -1 |  |  |  |  |  |  |  |
| 50 | 8 | 1 |  |  |  |  |  |  |  |
| 51 | 4 | 0 |  |  |  |  |  |  |  |
| 52 | 4 | 1 |  |  |  |  |  |  |  |
| 53 | 5 | 2 |  |  |  |  |  |  |  |
| 54 | 4 | 1 |  |  |  |  |  |  |  |
| 55 | 4 | 1 |  |  |  |  |  |  |  |
| 56 | 7 | 3 |  |  |  |  |  |  |  |
| 57 | 6 | 0 |  |  |  |  |  |  |  |
| 58 | 2 | 0 |  |  |  |  |  |  |  |
| 59 | 8 | 3 |  |  |  |  |  |  |  |
| 60 | 2 | 3 |  |  |  |  |  |  |  |
| 61 | 4 | 0 |  |  |  |  |  |  |  |
| 62 | 5 | 0 |  |  |  |  |  |  |  |
| 63 | 6 | 0 |  |  |  |  |  |  |  |
| 64 | 8 | 2 |  |  |  |  |  |  |  |
| 65 | 4 | 0 |  |  |  |  |  |  |  |
| 66 | 8 | 2 |  |  |  |  |  |  |  |
| 67 | 8 | 0 |  |  |  |  |  |  |  |
| 68 | 3 | 1 |  |  |  |  |  |  |  |
| 69 | 6 | 0 |  |  |  |  |  |  |  |
| 70 | 6 | 2 |  |  |  |  |  |  |  |
| 71 | 4 | 1 |  |  |  |  |  |  |  |
| 72 | 6 | 1 |  |  |  |  |  |  |  |
| 73 | 6 | 0 |  |  |  |  |  |  |  |
| 74 | 8 | 0 |  |  |  |  |  |  |  |
| 75 | 4 | 1 |  |  |  |  |  |  |  |
| 76 | 4 | 1 |  |  |  |  |  |  |  |
| 77 | 5 | 0 |  |  |  |  |  |  |  |
| 78 | 8 | 2 |  |  |  |  |  |  |  |
| 79 | 5 | 2 |  |  |  |  |  |  |  |
| 80 | 5 | 0 |  |  |  |  |  |  |  |
| 81 | 6 | 0 |  |  |  |  |  |  |  |
| 82 | 7 | 0 |  |  |  |  |  |  |  |
| 83 | 5 | 0 |  |  |  |  |  |  |  |
| 84 | 4 | 0 |  |  |  |  |  |  |  |
| 85 | 7 | 1 |  |  |  |  |  |  |  |
| 86 | 6 | 0 |  |  |  |  |  |  |  |
| 87 | 6 | 1 |  |  |  |  |  |  |  |
| 88 | 4 | 0 |  |  |  |  |  |  |  |
| 89 | 6 | 1 |  |  |  |  |  |  |  |
| 90 | 7 | 1 |  |  |  |  |  |  |  |
| 91 | 4 | 1 |  |  |  |  |  |  |  |
| 92 | 9 | 0 |  |  |  |  |  |  |  |
| 93 | 6 | 2 |  |  |  |  |  |  |  |
| 94 | 6 | 0 |  |  |  |  |  |  |  |
| 95 | 5 | 1 |  |  |  |  |  |  |  |
| 96 | 8 | 0 |  |  |  |  |  |  |  |
| 97 | 6 | 2 |  |  |  |  |  |  |  |
| 98 | 6 | 0 |  |  |  |  |  |  |  |
| 99 | 8 | 2 |  |  |  |  |  |  |  |
| 100 | 6 | 0 |  |  |  |  |  |  |  |
| 101 | 7 | 1 |  |  |  |  |  |  |  |
| 102 | 7 | 1 |  |  |  |  |  |  |  |
| 103 | 4 | 2 |  |  |  |  |  |  |  |
| 104 | 6 | 1 |  |  |  |  |  |  |  |
| 105 | 7 | 2 |  |  |  |  |  |  |  |
| 106 | 6 | 0 |  |  |  |  |  |  |  |
| 107 | 7 | 1 |  |  |  |  |  |  |  |
| 108 | 6 | 0 |  |  |  |  |  |  |  |
| 109 | 5 | 2 |  |  |  |  |  |  |  |
| 110 | 5 | 1 |  |  |  |  |  |  |  |
| 111 | 5 | 0 |  |  |  |  |  |  |  |
| 112 | 4 | 3 |  |  |  |  |  |  |  |
| 113 | 5 | -1 |  |  |  |  |  |  |  |
| 114 | 7 | 1 |  |  |  |  |  |  |  |
| 115 | 5 | 2 |  |  |  |  |  |  |  |
| 116 | 4 | -2 |  |  |  |  |  |  |  |
| 117 | 6 | 2 |  |  |  |  |  |  |  |
| 118 | 7 | 2 |  |  |  |  |  |  |  |
| 119 | 5 | 0 |  |  |  |  |  |  |  |
| 120 | 7 | 0 |  |  |  |  |  |  |  |
| 121 | 6 | 1 |  |  |  |  |  |  |  |
| 122 | 5 | 1 |  |  |  |  |  |  |  |
| 123 | 6 | 2 |  |  |  |  |  |  |  |
| 124 | 6 | 2 |  |  |  |  |  |  |  |
| 125 | 7 | 0 |  |  |  |  |  |  |  |
| 126 | 7 | 0 |  |  |  |  |  |  |  |
| 127 | 5 | 1 |  |  |  |  |  |  |  |
| 128 | 5 | 0 |  |  |  |  |  |  |  |
| 129 | 10 | 0 |  |  |  |  |  |  |  |
| 130 | 7 | 0 |  |  |  |  |  |  |  |
| 131 | 5 | 1 |  |  |  |  |  |  |  |
| 132 | 5 | 2 |  |  |  |  |  |  |  |
| 133 | 5 | 0 |  |  |  |  |  |  |  |
| 134 | 6 | 3 |  |  |  |  |  |  |  |
| 135 | 2 | 2 |  |  |  |  |  |  |  |
| 136 | 5 | 0 |  |  |  |  |  |  |  |
| 137 | 7 | 1 |  |  |  |  |  |  |  |
| 138 | 5 | 0 |  |  |  |  |  |  |  |
| 139 | 7 | 0 |  |  |  |  |  |  |  |
| 140 | 5 | 1 |  |  |  |  |  |  |  |
| 141 | 5 | 1 |  |  |  |  |  |  |  |
| 142 | 6 | 2 |  |  |  |  |  |  |  |
| 143 | 6 | 2 |  |  |  |  |  |  |  |
| 144 | 10 | 3 |  |  |  |  |  |  |  |
| 145 | 6 | 1 |  |  |  |  |  |  |  |
| 146 | 4 | 2 |  |  |  |  |  |  |  |
| 147 | 8 | -1 |  |  |  |  |  |  |  |
| 148 | 6 | -1 |  |  |  |  |  |  |  |
| 149 | 8 | 1 |  |  |  |  |  |  |  |
| 150 | 4 | 0 |  |  |  |  |  |  |  |
| 151 | 4 | 1 |  |  |  |  |  |  |  |
| 152 | 5 | 2 |  |  |  |  |  |  |  |
| 153 | 4 | 1 |  |  |  |  |  |  |  |
| 154 | 4 | 1 |  |  |  |  |  |  |  |
| 155 | 7 | 3 |  |  |  |  |  |  |  |
| 156 | 6 | 0 |  |  |  |  |  |  |  |
| 157 | 2 | 0 |  |  |  |  |  |  |  |
| 158 | 8 | 3 |  |  |  |  |  |  |  |
| 159 | 2 | 3 |  |  |  |  |  |  |  |
| 160 | 4 | 0 |  |  |  |  |  |  |  |
| 161 | 5 | 0 |  |  |  |  |  |  |  |
| 162 | 6 | 0 |  |  |  |  |  |  |  |
| 163 | 8 | 2 |  |  |  |  |  |  |  |
| 164 | 4 | 0 |  |  |  |  |  |  |  |
| 165 | 8 | 2 |  |  |  |  |  |  |  |
| 166 | 8 | 0 |  |  |  |  |  |  |  |
| 167 | 3 | 1 |  |  |  |  |  |  |  |
| 168 | 6 | 0 |  |  |  |  |  |  |  |
| 169 | 6 | 2 |  |  |  |  |  |  |  |
| 170 | 4 | 1 |  |  |  |  |  |  |  |
| 171 | 6 | 1 |  |  |  |  |  |  |  |
| 172 | 6 | 0 |  |  |  |  |  |  |  |
| 173 | 8 | 0 |  |  |  |  |  |  |  |
| 174 | 4 | 1 |  |  |  |  |  |  |  |
| 175 | 4 | 1 |  |  |  |  |  |  |  |
| 176 | 5 | 0 |  |  |  |  |  |  |  |
| 177 | 8 | 2 |  |  |  |  |  |  |  |
| 178 | 5 | 2 |  |  |  |  |  |  |  |
| 179 | 5 | 0 |  |  |  |  |  |  |  |
| 180 | 6 | 0 |  |  |  |  |  |  |  |
| 181 | 7 | 0 |  |  |  |  |  |  |  |
| 182 | 5 | 0 |  |  |  |  |  |  |  |
| 183 | 4 | 0 |  |  |  |  |  |  |  |
| 184 | 7 | 1 |  |  |  |  |  |  |  |
| 185 | 6 | 0 |  |  |  |  |  |  |  |
| 186 | 6 | 1 |  |  |  |  |  |  |  |
| 187 | 4 | 0 |  |  |  |  |  |  |  |
| 188 | 6 | 1 |  |  |  |  |  |  |  |
| 189 | 7 | 1 |  |  |  |  |  |  |  |
| 190 | 4 | 1 |  |  |  |  |  |  |  |
| 191 | 9 | 0 |  |  |  |  |  |  |  |
| 192 | 6 | 2 |  |  |  |  |  |  |  |
| 193 | 6 | 0 |  |  |  |  |  |  |  |
| 194 | 5 | 1 |  |  |  |  |  |  |  |
| 195 | 8 | 0 |  |  |  |  |  |  |  |
| 195 | 6 | 2 |  |  |  |  |  |  |  |
| 197 | 6 | 0 |  |  |  |  |  |  |  |
| 198 | 8 | 2 |  |  |  |  |  |  |  |
| 199 | 6 | 0 |  |  |  |  |  |  |  |
|  |  |  |  |  |  |  |  |  |  |
